# Defining the effect and mediators of two knowledge translation strategies designed to alter knowledge, intent and clinical utilization of rehabilitation outcome measures: a study protocol [NCT00298727]

**DOI:** 10.1186/1748-5908-1-14

**Published:** 2006-07-04

**Authors:** Joy C MacDermid, Patty Solomon, Mary Law, Dianne Russell, Paul Stratford

**Affiliations:** 1 School of Rehabilitation Science, McMaster University, 1400 Main St. West, IAHS-403, Hamilton, Ontario, L8S 1C7, Canada; 2 Hand and Upper Limb Centre Clinical Research Laboratory, St. Joseph's Health Centre, 268 Grosvenor St., London, Ontario, N6A 3A8, Canada

## Abstract

**Background:**

A substantial number of valid outcome measures have been developed to measure health in adult musculoskeletal and childhood disability. Regrettably, national initiatives have merely resulted in changes in attitude, while utilization remains unacceptably low. This study will compare the effectiveness and mediators of two different knowledge transfer (KT) interventions in terms of their impact on changing knowledge and behavior (utilization and clinical reasoning) related to health outcome measures.

**Method/Design:**

Physical and occupational therapists (n = 144) will be recruited in partnership with the national professional associations to evaluate two different KT interventions with the same curriculum: 1) Stakeholder-Hosted Interactive Problem-Based Seminar (SHIPS), and 2) Online Problem-Based course (e-PBL). SHIPS will consist of face-to-face problem-based learning (PBL) for 2 1/2 days with outcome measure developers as facilitators, using six problems generated in consultation with participants. The e-PBL will consist of a 6-week web-based course with six generic problems developed by content experts. SHIPS will be conducted in three urban centers in Canada. Participants will be block-allocated by a minimization procedure to either of the two interventions to minimize any prognostic differences. Trained evaluators at each site will conduct chart audits and chart-stimulated recall. Trained interviewers will conduct semi-structured interviews focused on identifying critical elements in KT and implementing practice changes. Interviews will be transcribed verbatim. Baseline predictors including demographics, knowledge, attitudes/barriers regarding outcome measures, and Readiness to Change will be assessed by self-report. Immediately post-intervention and 6 months later, these will be re-administered. Primary qualitative and quantitative evaluations will be conducted 6-months post-intervention to assess the relative effectiveness of KT interventions and to identify elements that contribute to changing clinical behavior. Chart audits will determine the utilization of outcome measures (counts). Incorporation of outcome measures into clinical reasoning will be assessed using an innovative technique: chart-stimulated recall.

**Discussion:**

A strategy for optimal transfer of health outcome measures into practice will be developed and shared with multiple disciplines involved in primary and specialty management of musculoskeletal and childhood disability.

## Background

Patient-oriented health outcomes are key to assessing health care in chronic illness. Chronic disability, as a result of adult musculoskeletal or childhood disorders, is profound and comprises a large component of practice for a variety of health care providers. Musculoskeletal diseases of adulthood are the leading cause of long-term disability in Canada, accounting for roughly one-third of the country's long-term disability costs [1]. Childhood disorders also account for a large percentage of disability treatment costs with 1 in 12 children now considered disabled; increasing rates are attributable to improvements in medical care that save more compromised children, broader definitions of disability, and a greater willingness to report handicaps [2]. Due to the chronic nature of these disorders, treatment is focused on minimizing disability and improving quality of life. Standardized measurement of the impact of interventions on these health outcomes is fundamental to advancing clinical practice and research.

The current use of patient-oriented outcome measures in research and practice is deficient, despite the fact that health care professionals recognize the importance of measuring health outcomes and efforts have been made to transfer available knowledge into practice. These efforts include national initiatives by the professional associations of both occupational therapists (OT) and physical therapists (PT), traditional workshops [3], published editorials [4], scientific articles [5-16], textbooks [17], professional association endorsements, and promotion of an outcomes database. While agreement with the need for outcome measures is consistently high, utilization remains low across professional groups dealing with these chronic problems, such as Rehabilitation [18], Rheumatology [7,19], and Orthopaedic Surgery [20]. Rehabilitation is commonly performed by PTs and OTs in a variety of practice settings. As few knowledge transfer studies have included these providers, we decided to focus on PTs and OTs for this study. The needs have been well established in this area, and the investigators have established partnerships with the associated national professional associations who will facilitate the current project and arising national KT initiatives.

The deficiency in current practice indicates a failure to implement effective knowledge transfer, and systematic reviews confirm that KT is based on inadequate evidence. The current failure to implement health status measures into practice is not unexpected; reviews of available evidence suggest that traditional dissemination/continuing education has little substantive impact on clinical behavior. A large body of evidence has been developed on the impact of continuing education. Studies of high quality have been synthesized in systematic reviews [21-23]. These reviews have focused on physician behavior, in particular, concrete medical outcomes such as prescription practices that are quite different from rehabilitative interventions. Nevertheless, they do provide some indication of KT approaches that might be used in other areas where evidence is lacking.

Separate reviews have addressed printed education materials, educational outreach visits, local opinion leaders, and continuing education workshops/meetings [21,22,24-27]. Each strategy was shown to lead to a measurable change, although the impact of printed materials was small and of uncertain clinical significance [27]. Neither audit and feedback [28-30] nor conferences [31] made substantial change in practice, with larger effects occurring through occasional outreach visits and use of opinion leaders [32]. Educational outreach visits were investigated in 18 randomized trials that were independently reviewed by two researchers [26] and shows that outreach with supporting materials was more effective than no intervention. Again, physician-prescribing practices were the most common target behaviour. In five separate trials, it was shown that outreach visits with social marketing were most effective when high prescribers were targeted [33-35]. However, little evidence addresses the optimal timing or frequency of outreach or whether changes in practice are maintained over time. A single study [36] included 2-year follow-up and demonstrated that new prescribing behaviours were maintained over time.

Continuing education meetings and workshops were addressed in 32 studies that were judged to be of moderate to high quality and included 2995 health professionals, usually physicians [22]. Interactive workshops were shown to have moderate to large effects in six studies and small effects in four. Combinations of workshop and didactic presentation also were effective, showing moderate or large effects in 12 studies and small effects in seven [22]. Seven studies addressing didactic presentations showed no significant impact. It was suggested that didactic presentations might improve knowledge without impacting on practice, whereas small group discussion and practice might improve skills/behavior. Unfortunately, only a single trial made this comparison and it had inconclusive results. Cochrane reviewers suggested that further (high-quality) studies are required, and they should focus on interactive workshops. They also suggested that future studies should use qualitative processes to clarify how specific attributes of workshops contribute to effects on professional practice [22].

There is a specific lack of knowledge on the impact of knowledge transfer on complex clinical decision-making. The majority of intervention trials attempting to change clinical behavior have focused on the prescribing practices of physicians, limiting the generalizability to clinical practices used to manage chronic musculoskeletal or childhood disability problems. Management of chronic conditions requires that health care professional deal with multi-factorial disability issues by selecting multi-level customized interventions. It is more difficult to assess how KT impacts on clinical decision-making in this situation, as compared to monitoring adherence to prescription recommendations. Beggs and Sumison [37] presented a model that incorporated multi-level evaluation of the long-term benefits of continuing education within a Northern Outreach Program for PT and OT. They proposed a 4-stage model of evaluation. Stage 1 involves participant evaluation of the event. Stage 2 evaluates the affective, cognitive, and psychomotor changes that participants experience as a result of the event; this typically requires a pre-test and post-test of attitudes, knowledge, or specific skills. In Stages 3 and 4, higher levels of evaluation are incorporated. Stage 3 evaluates the extent to which programs change the behavior of the clinician within their practice and requires chart audits and observations. Stage 4 focuses on the client and requires evaluation of the efficiency, effectiveness, adequacy and appropriateness of care and its impact on resultant health outcomes.

We know from surveys of orthopedic practice [18] that the use of standardized health outcome measures is low. Conversely, within pediatric rehabilitation utilization levels are higher, but therapists reported difficulty in selecting and applying available outcome measures appropriately (pilot work, publication under review). It is clear that evaluation of knowledge transfer should measure changes in knowledge, intent, and behavior, but also determine how new knowledge is incorporated into clinical decision-making.

Systematic reviews have highlighted the need to better understand the mediators of knowledge transfer, and previous work has established that a variety of factors may influence the effects of KT [38,39]. However, the mediators are usually only addressed as secondary issues, and few high-quality studies or literature synthesis have been conducted. Prior knowledge, education, and age have been considered as demographic predictors. We will evaluate the role of these previously studied predictors. However, we also wish to identify unknown predictors. To fully address KT mediators, it is important to have an in-depth understanding of responses to knowledge transfer; this requires qualitative research that identifies and characterizes the elements that facilitate or obstruct KT. It is our belief that it is important to identify mediators that could be used to maximize KT effectiveness using a proactive approach. 'Readiness to Change,' also called the Transtheoretical Model, incorporates features of a variety of behavior models to describe the stages of change. It has been used in addiction, health promotion, organizational change, and professional practice literature, most commonly health behavior applications [40,41]. More recently, some have suggested that Readiness to Change may provide a greater depth of understanding of how participants respond to knowledge transfer [42]. Specifically, these investigators used a Readiness to Change questionnaire to evaluate how KT affected intent and action to a short course on knowledge transfer. The Readiness to Change model suggests that change in behavior is modulated by a person's readiness to make changes at the time the information is provided [40,41,43]. In other words, "*the right information and the right process – at the right time.*" The stages are: **Precontemplation **(uninformed about the need for change, uninterested in changing behavior), **Contemplation **(thinking about change in the near future), **Preparation **(ready to make a change in the next month), **Action **(implementing a specific action plan), and **Maintenance **(continuation of desirable actions). The model developers [44-46] and subsequent studies [40,43,47-51] suggest that categorizing people in stages allows one to customize messages and strategies specific to the participant's stage. This concept has not been applied to KT, but if we demonstrate that readiness to change mediates responses in this study, it will provide a promising approach to customize knowledge transfer to users. We will use the qualitative component of the study to understand the decisional balance inherent in the Transtheoretical Model.

Knowledge transfer interventions should bring knowledge into action. Constructivist principles recognize that knowledge is, "not a thing to be sent, but a fluid set of understandings shaped by both those who originate it and by those who use it" [52]. The user is seen as an active problem solver and a constructor of his/her own knowledge rather than a receptacle of information [52]. Clinicians must be able to use outcome measures within a valid and practical framework. Knowledge transfer strategies that engage researchers and clinicians to resolve these competing requirements may be more successful in facilitating the use of outcome measures. The possession of knowledge does not mean that it will be used. The need to go beyond dissemination that simply reflects successful distribution towards effective dissemination that requires use of the information has been emphasized [53]. Huberman [52] differentiated *conceptual use of knowledge*, which is characterized by changes in knowledge, understanding or attitude, from *instrumental use *that includes changes in behavior and practice. Practice surveys indicate both conceptual and instrumental knowledge deficits exist in musculoskeletal and pediatric practice [54]. Knowledge transfer interventions must target and assess both.

McMaster University has a worldwide reputation for educational innovation and problem-based learning (PBL). PBL is an ideal pedagogical strategy for facilitating knowledge transfer. Research on memory suggests memory and learning can be enhanced by: maximizing the positive effects of context by closely matching the learning and clinical environments, enhancing meaning by activating relevant prior knowledge, using educational activities that require the participants to elaborate on their information, and ensuring that new knowledge is used repeatedly in a number of different contexts [55]. The elaboration of information that occurs in tutorial discussion, the use of problems to match new knowledge to the clinical context, and the activation of prior knowledge have been recognized as active components of PBL [56]. Therefore PBL helps in the contextualization of knowledge and in the application of knowledge, which are key components of the CIHR knowledge transfer model (listed as KT3 and KT5 by CIHR).

The rationale for a PBL approach to knowledge transfer is based on solid evidence of adult learning and the effects of PBL [58,59]. This work has shown that PBL is not more effective in acquiring knowledge, but *is *more effective in generating a life-long learning approach where learners become more self-directed in fulfilling their personal learning issues and applying acquired knowledge to problems [58]. This may be the critical component needed in KT, where users must incorporate new knowledge into clinical practice and resolve inherent barriers before implementing change.

Research on KT strategies suggests that the strategy must be tailored to the types of decisions that clinicians face and to the environments in which they work [60]. It is important to consider organizational and political factors that may influence decisions to incorporate new knowledge [60]. Therefore, the curricular design of both knowledge transfer strategies will incorporate contextual learning principles within a PBL framework. Research on both adult education and on effective knowledge transfer suggests that passive learning is ineffective and that interactive strategies are necessary to be successful [60]. While both the interventions will be problem-based and involve interaction, the strategies will differ in the delivery mode. One strategy will incorporate face-to-face PBL, whereas the other will be internet-based. Hence, the nature of interaction will be quite different between delivery modes.

Traditionally, PBL is highly dependent on face-to-face interaction. Effective knowledge transfer is supported through these types of interactions, particularly if associated with an opinion leader [32]. The opportunity for meaningful engagement between researchers developing outcome measures and clinicians using them through a traditional PBL process should augment KT that supports "instrumental use." There is a strong body of evidence supporting the effectiveness of traditional face-to-face PBL education that suggests it will assist clinicians to acquire higher level reasoning, incorporate newly acquired information, and address barriers to implementing new outcome measures [56,59,61]. It is unclear whether the inherent value of face-to-face interaction with developers outweighs the time constraints of this form of knowledge transfer. Research on PBL indicates that learners are initially inefficient and stressed with this new approach to learning [62]. While the learning curve is steep, it is not unattainable. Participants in our pilot study reported that the PBL was time-consuming, but valued.

A rapidly evolving mode of accessing information and continuing education is through the use of the Internet. Online course work has proliferated at a pace well beyond the capacity of educational/KT researchers to study its effectiveness or implications. While theoretical papers on online learning have laid out the pedagogical issues, few high-quality research studies have addressed learning outcomes in a quantitative way. A recent study reviewed all studies indexed on Medline that addressed Internet-based medical education [63] to determine the extent of formal evaluation. Of 85 studies, 55 merely described the program and provided no evaluation. Of the remaining 31 studies, 81% evaluated participant satisfaction, 52% evaluated learning outcomes, and only 6% evaluated change in clinical practice behaviors.

Despite the low level of evidence surrounding online professional education, there is a rationale for this approach. One potential benefit is that participants can access information/course work asynchronously. If participation in face-to-face PBL is a significant barrier to busy clinicians, online interaction might be preferable. There are advantages to online learning that may promote knowledge transfer. For example, online learning allows for increased time for reflection and synthesis [64,65] and provides increased time to develop the ability to organize thoughts when problem-solving collaboratively [64]. Online learning and online forums also are thought to promote critical thinking and problem-solving in a collaborative environment [66]. Despite these potential benefits, few studies have specifically examined online PBL. Dennis [67] compared online PBL and face-to-face PBL and found there was no difference in learning outcomes. However, the online groups spent more time on learning, suggesting that this process was less efficient. Chan et al. [68] randomized family physicians to either Internet-based PBL or a control group (Internet content without PBL) and found no difference in knowledge. However, the sample was small (n = 23). In a qualitative study, Valaitis et al. (2005) examined health science students' perceptions of online PBL. The results showed that students valued the flexibility of online learning and felt it enhanced their ability to deeply process content, but they had initial difficulties adapting to an online environment and perceived a heavy workload. Given the current state of practice and knowledge, we propose to evaluate two KT approaches to implanting knowledge on outcome measures.

## Purpose

### Primary objective of the study

This study will evaluate the effectiveness of two innovative knowledge transfer interventions using a quasi-experimental, mixed-methods research design. Specific objectives include:

1. To determine the relative effectiveness of a Stakeholder-Hosted Interactive Problem-based Seminar (SHIPS) and Online Problem-Based tutorials (e-PBL) in changing knowledge, utilization, and integration of knowledge in clinical decision-making.

### Secondary objectives

1. To identify the key elements of SHIPS and e-PBL that engage participants in KT and assist them in addressing barriers to change;

2. To determine whether clinicians exhibit a decisional balance and spectrum of behaviors consistent with the Transtheoretical Model of (Readiness to) Change; and

3. To determine the relative importance of potential predictors of change, including characteristics of clinicians (years of practice, highest degree, Readiness To Change), practice settings (practice type, caseloads, years of experience), and how they affect knowledge acquisition and implementation following KT interventions.

## Method/Design

This study will implement two knowledge transfer interventions at three sites across Canada and determine the intervention effectiveness and its mediators using a mixed qualitative quantitative approach.

### Rationale for a mixed-methods approach

Cochrane reviewers have suggested that a mixed-method approach is required to understand how to change clinical behaviour [22]. This study has a strong quantitative foundation based on specific research questions that will be answered using validated instruments to assess KT outcomes. However, a qualitative approach is needed to augment this information. A qualitative approach will be used to elucidate the specific key elements that enhance or obstruct the effectiveness of these two new KT approaches and to understand the decisional balance that underlies the process of changing clinical behavior in response to knowledge transfer.

### Rationale for phased implementation in three cities

We have recruited three sites across Canada. At each site a clinical partner "host" will assist in recruitment of participants and local organizations. We felt national representation was important to provide generalizable results and to insure that this project facilitates KT networks that will support future national initiatives for broader implementation. We specifically did not use Hamilton, as we felt it was "contaminated" by numerous prior activities conducted by study investigators. The Kitchener-Waterloo site will be the alpha site, with the second wave of KT intervention taking place in Calgary and Halifax. The phased approach has several advantages. For instance, it allows us to train the research assistants from the Calgary and Halifax areas in a central location. Based at McMaster University, the project coordinator will have the primary responsibility for project coordination, with site research assistants sharing site organization and local chart audit evaluation. These research assistants will come to the first KT site to undergo standardized training on the chart audit and chart-stimulated recall procedures. This will insure they have a comprehensive understanding of the interpretation of responses during the chart-stimulated recall. Their orientation will consist of training on the theory and methods of chart-stimulated recall, participation in both KT strategies, and observation of the chart audit (use and stimulated recall) conducted by study investigators at the alpha site. This will insure consistency across the three sites. A further advantage of the phased approach is that we will be able to maximize the value of our qualitative component evaluating the *process *of KT by making changes to qualitative probes as indicated by alpha site results. That is, we will be able to commence the iterative qualitative analysis that will inform further qualitative data collection and analysis, providing an enhanced understanding of how changes in clinical behavior are motivated.

### Subjects

#### Recruitment

Participants will be recruited from the surrounding clinics/organizations through existing communications links (e.g., professional newsletters, listservs, and local meetings) and through letters of invitation distributed to eligible clinics in the three cities. In addition, the professional associations have agreed to assist with recruitment though websites and advertisements. Based on our previous projects and pilot work, we anticipate high levels of participation.

#### Inclusion/exclusion criteria

A valid license to practice physical or occupational therapy, and ability to communicate in English is required. Volunteers will be required to complete a knowledge pre-test in the format of a multiple-choice questionnaire. Those who are already knowledgeable, as determined by a score of 75% or greater, will be excluded to avoid ceiling effects (pilot work suggests this will be rare).

#### Sample size requirements

Given that this study is a mixed-methods design, the sample size was based on the quantitative analyses as these have larger sample size requirements. Sample size estimation was based on detecting an effect of 0.50 between groups on any of the three aspects of outcome (knowledge, utilization, and integration into clinical reasoning). Assuming Type I error = 0.05 (2-tailed); Type II error = 0.80; Effect size = 0.50, the sample size required per group = 64. The sample size required for two-comparison groups = 128 and accounting for a 10% dropout = 128/0.9 = 142. We anticipate low dropouts given the priority of continuing education by both professions. We will round our sample size up to 144 to provide a number equally divided between three sites, requiring 48 per site. Based on the need to allocate participants in blocks to interventions and to balance professions and clinical areas evenly, we expect to accommodate 24 participants per intervention group, per site. These will consist of three tutorial groups of eight therapists/groups. Given the distribution of practice patterns in rehabilitation, we expect two groups on orthopedics and one group on pediatric practice at each location. Groups will be formed according to practice settings to insure that the stakeholders can develop "problems" that simulate their own clinical settings/populations.

#### Group assignment procedure

A randomized design is usually the most rigorous, allowing for control of known and unknown confounders. In this case, it is not the most appropriate design strategy and we have selected a quasi-experimental approach. Research design methodologists have indicated that attaining an equal distribution of confounders in small samples via randomization, such as that required for the present study, is unreliable. Therefore we will use a non-randomized allocation procedure called minimization, which places participants in intervention groups to minimize the differences across key predictors [69-71]. We have identified pre-test scores, years of practice, practice area (urban/rural), and practice type (PT/OT) as the key predictors. Minimization across key predictors will balance prognostic variables and result in more valid comparisons [71]. Subjects will be allocated using minimization within orthopedic and pediatric groups at each site. At each site the pool of subjects will be allocated minimizing difference by: creating pair groupings based on professional training (PT/OT), matching area practice and then most similar pre-test scores, and, finally, by minimizing years of practice. We then will conduct descriptive analyses of group similarities and test whether we can optimize groups' consistency by reallocation of assignment. When this process is complete, subjects at each site will be informed of their assignment.

#### Interventions

There will be two knowledge transfer interventions with different delivery methods. The learning objectives, content covered, and number of contact hours will be similar for both. The KT will address how to: select health status measures for clinical practice, score/interpret results, incorporate measures into clinical reasoning, and recognize and address personal and organizational barriers and facilitators of change.

#### Stakeholder-Hosted Interactive Problem-based Seminars (SHIPS)

The SHIPS will consist of a 2 1/2-day interactive PBL sessions with 10 hours of contact/tutorial time and 15–20 hours of facilitated independent group work that will focus on application of learned concepts. Consistent with a problem-based philosophy, small groups of clinicians will participate in interactive sessions facilitated by a faculty tutor. The faculty tutor will be a developer of outcome measures, an expert facilitator in PBL, and one of the study investigators. The SHIPS' knowledge transfer strategy is based on evidence establishing the importance of using opinion leaders with scientific and professional credibility [32], and will be operationalized using our experience in PBL as a method of providing contextualized learning. Six "problems" will be generated by faculty through a consultative process with the participants prior to the sessions. Problems will be generated to reflect the established curriculum, with a problem that represents the practice characteristics and issues expressed by participants. Participants will conduct this process four weeks prior to the SHIPS intervention and will receive the curriculum, course objectives, and a recommended reading list one week prior to attendance at the SHIPS.

#### Online Problem-based Course (e-PBL)

The web-based intervention will consist of six weekly e-PBL sessions with 10 contact hours and 10–15 facilitated independent learning activities. Six generic problems will be developed by the study investigators to meet the curriculum objectives. The e-PBL will be delivered over a relatively short period (six weeks) as previous research demonstrated a large drop-out rate with 14 weeks [72]. Sessions will be facilitated and monitored by a faculty member who is familiar with web-based instruction, PBL, and has expertise in outcome measures. Study investigators will ensure visibility through their participation in online chats and content development, but the operation of the online delivery will be managed by an educator with expertise in online delivery. The consistent components of each session will be a problem generated by the faculty to represent key concepts regarding outcome measures, session objectives, a recommended reading list, and discussion questions. Discussion questions will be addressed through asynchronous online chat amongst participants, as facilitated by faculty tutors.

### Study measures

#### Baseline measurement of eligibility, status, and potential KT predictors

Participants will be pre-screened to ensure inclusion criteria. Eligible participants will then complete a baseline knowledge pre-test (to avoid ceiling effects/lack of responsiveness). Participants also will complete a baseline information questionnaire to collect demographic data, practice patterns, and educational background. Survey measures of knowledge and behavior will be administered. This data collection also will include a measurement of the therapist's intent to use outcome measures, their general level of research utilization, and their readiness to change. This scale evaluating Readiness to Change [40,41,73] was developed to reflect the core elements of the five stages of change, but was specifically applied to changing clinical practice. Although all participants will have agreed to allocation during informed consent, they will be asked their preference with respect to e-PBL and SHIPS so that post-hoc analyses can determine the importance of educational preference as a mediator of response.

#### Post-intervention evaluations of KT impact

##### 1) Knowledge

The screening multiple-choice test will form the baseline knowledge score. Alternate forms of this test will be devised for pediatric and musculoskeletal populations; test content will be mapped to the curriculum objectives. A bank of questions reflecting the key knowledge curriculum will be developed, and participants will be provided with alternate forms for pre- and post-test evaluations to minimize recall bias as a potential reason for score inflation.

##### 2) Utilization

Chart audit will be used to measure utilization of outcome measures. In many situations, chart audit does not accurately represent the content of a clinical interaction because not all information is recorded [74]. However, in our case the reverse is true. Specific self-report forms are required to administer outcome measures and will provide direct evidence of utilization. Charts will be selected for a chart audit procedure as follows: a) one day from each participant's previous month of practice will be selected randomly, b) a list of patients seen on that day will be generated, and c) five patient charts will be selected randomly from that daily list. Using a standardized data extraction form, the entire chart record will be audited to determine the total numbers of outcome measures used, the frequency of use, the timing of use (i.e., every session, at evaluation and discharge), and the specific outcome measures used. In addition, it will be recorded whether scales were scored correctly, and whether the scales were specifically mentioned in goal setting or discharge planning.

##### 3) Integration of knowledge

The integration of knowledge into clinical decision-making is more complex than measures of utilization and, hence, more difficult to measure. However, as the ultimate purpose of new knowledge is to improve the quality of care, evaluation of how clinicians use new information to make decisions is critical. Simple measures of the use of concrete behaviors – prescription practices or completion of outcome scales – provide information on whether practitioners are receptive to changing their behavior. However, these measures do not provide insights about whether these altered clinical behaviors are integrated into higher-level clinical reasoning. As reviewed above, these higher-level evaluations are rarely incorporated into KT evaluations [63]. Chart-stimulated recall [75-77] is an evaluation method that combines personal interview and chart audit to engage participants in a reflective discussion on these deeper levels of cognitive reasoning. A trained evaluator draws inferences from the information to rate the clinician's behavior on a variety of items that reflect clinical reasoning and competency in the area of interest. This method was originally developed at McMaster University to evaluate competence in medical practitioners [76], and it has been shown to be a valid process in this population [75,76,78], as well as amongst occupational therapists [77].

The chart-stimulated recall form must be developed specifically for the competencies being evaluated. The competencies evaluated in this study will be the core curriculum about outcome measurements, with an emphasis on their application to clinical reasoning. This focus includes the clinician's ability to provide: a rationale for why specific outcome measures were selected for specific patients, an understanding of the correct application of the scale, an ability to use the obtained score to determine disability and prognosis, and the ability to set rehabilitation goals based on disability scores, including clear parameters for the expected change in scores following intervention. Chart-stimulated recall responses are scored on a seven-item scale that reflects the extent of competency [77]. The staged process of the study design will enable high-quality evaluations during chart-stimulated recall by allowing evaluators from Eastern and Western Canada to participate in Phase 1 of the project as a means of gaining greater consistency between raters.

The chart-stimulated recall will be conducted by a single trained research assistant assigned to each location. Two of the five charts selected for chart audit will be randomly selected for chart-stimulated recall. The interviewer will ask questions in a semi-structured format that requires specific responses from the therapist, explaining the content and clinical reasoning used for the two patients whose records are used to evaluate the core competencies being tested. The answers are scored on a seven-point scale. The spectrum of information included in the chart-stimulated recall analysis will include all intake assessments, progress notes, and discharge records for a specific patient. A detailed manual on the types of responses required will be developed in conjunction with curriculum development. Chart-stimulated recall will provide a quantitative assessment of the clinical reasoning used with respect to the use of outcome measures in managing specific patients.

## Analyses

### Quantitative analyses

All data will be double-entered in SPSS 14.0. Descriptive analyses will be conducted, including checking for outliers, normality testing, and univariate correlations. The first primary analysis (Objective 1) will be a two-way repeated measures analysis of variance to determine primary unadjusted differences in absolute scores over time, and between groups for each of the three primary outcome measures: knowledge, utilization, and chart-stimulated recall scores. An analysis of covariance will be used to compare these same effects adjusting for baseline knowledge score, Readiness to Change, years of practice, and educational preference. These analyses across the e-PBL and SHIPS groups will determine the relative effectiveness of these two alternative knowledge transfer choices. Effect sizes and their 95% confidence intervals will be calculated to determine whether there are differential impacts on change in knowledge, utilization, and integration of knowledge between the two different KT approaches. For the secondary research question (Objective 2.3) on the relative importance of KT predictors, a multiple linear regression [79,80] will be used to develop models of how years of practice, educational background, caseload characteristics, educational preferences, or Readiness to Change predict changes in knowledge, utilization, or integration following knowledge transfer interventions, with KT method as a covariate.

### Qualitative assessment/evaluation

The qualitative assessment will enable us to identify the key elements of SHIPS and e-PBL that engage participants in knowledge transfer and any associated facilitators/barriers to change (Objective 2.1.). We also will identify the specific pros and cons of change so that we can determine the decisional balance (Objective 2.2). From the qualitative and quantitative findings we will be able to ascertain whether the spectrum of behaviors and decisional balance is consistent with the Transtheoretical Model of Change. We will identify in detail the therapists' experiences in incorporating outcome measures into their practice and their overall perceptions of the effectiveness of the specific KT intervention. We will document which components of KT strategies are conducive to knowledge transfer and which present barriers. We will also specifically probe participants on the decisional balance for undergoing change in clinical practice. At study entry, participants will be asked if they would be willing to participate in a short (10–15 minute) baseline and longer (15–30 minute) follow-up telephone interview. The baseline interview will emphasize the facilitators/barriers to participating in the KT intervention and issues affecting their decisional balance. The post-intervention interviews will emphasize valued elements/barriers experienced with each KT intervention, facilitators/barriers to change, and the impact of knowledge transfer.

We previously successfully used telephone interviews to interview participants who encompass large geographical distances. We will purposively select 30–40 interviewees from those who volunteer. Interviewees will be balanced by type of intervention, area of practice (musculoskeletal and pediatric), profession (OT and PT), and geographic location of practice (West, East, Central).

Interviews will be conducted by a trained interviewer, knowledgeable in qualitative methods, who is unknown to the participants. Interviews will be audiotaped and transcribed verbatim. Content analyses of the interview transcripts will proceed using an open coding technique [81] with the assistance of a qualitative software program (N6). [N6 is a tool for code-based inquiry and searching which is particularly useful for working with large amounts of data in a team environment.] The analysis will consist of a line-by-line review of the transcripts to develop codes related to the specific comments and experiences of the therapists. Initially, three transcripts will be reviewed independently by three team members. They will meet to discuss and reach agreement on the codes. Once agreement on codes is reached, the remaining transcripts will be reviewed to identify similarities, patterns, and common sequences. Categories or themes related to the patterns, processes, and commonalities will emerge through this process [81, 82, 83]. The themes will then be used to develop an in-depth description of the participants' experiences and perceptions. The data collection and analysis will be conducted iteratively. We will initially interview and analyze the data from 30 participants. We will continue recruitment to a maximum of 40 participants or until saturation of the data is achieved (94;95) [81, 82]. The following key questions will be utilized to frame semi-structured interviews:

#### Baseline probes

• What are the attitudes with respect to acquiring, integrating, and contextualizing new knowledge?

• What are the pros/cons of changing clinical practice?

• What are the organizational and personal barriers and facilitators to participation in KT?

#### Post-intervention probes

• What are the key elements of SHIPS and E-PBL knowledge transfer (positive and negative influences)?

• What are the organizational and personal barriers and facilitators to changing clinical behavior?

• What are the strategies that assist with changing practice?

• What aspects of the decisional balance change in response to KT?

• What are the ongoing needs required to build on the impact of the KT?

## Discussion

### Knowledge impact

Our primary purpose is to better understand these two novel approaches to knowledge transfer. We choose outcome measures as a KT target for substantial reasons. Firstly, the knowledge base of standardized disability measures is strong, and there are a number of studies that demonstrate that this knowledge has not been implemented into clinical practice. Therefore, we can expect a substantial improvement in clinical practice if knowledge uptake is facilitated through this study. In terms of KT research design, this study provides an ideal model because it is possible to make rigorous measurements of knowledge, utilization, and clinical reasoning, providing deeper understanding of knowledge transfer. Finally, we felt that the generalizability of our findings would be broad as the KT issues identified in rehabilitation practice also have been reported across a number of professions and practice settings [7,18-20] dealing with patients who have chronic disability related to musculoskeletal or pediatric disorders. Finally, musculoskeletal and pediatric disorders account for increasing amounts of disability in the population, and it is imperative that health care providers implement outcome measures to assure effective and efficient use of future health care resources.

## Competing interests

The author(s) declare that they have no competing interests.

## Authors' contributions

J MacDermid proposed the general research question. All authors contributed to the development of the specific research question and defining study objectives and methods. JM identified study outcome measures and wrote the proposal; P Stratford conducted sample size calculations; and P Solomon developed qualitative analyses. M Law and D Russell conducted pilot work. All authors revised and approved all aspects of the final study protocol.
